# Evaluation of Foveal Vasculature by Optical Coherence Tomography Angiography after Pan-Retinal Photocoagulation versus Intravitreal Anti-VEGF Injections

**DOI:** 10.18502/jovr.v19i3.13622

**Published:** 2024-09-16

**Authors:** Hamid Riazi-Esfahani, Amin Ahmadi, Reza Sadeghi, Masoud Mirghorbani, Fariba Ghassemi, Mohammad Zarei, Hassan Khojasteh, Nikoo Bayan, Hooshang Faghihi, Elias Khalili Pour, Ahmad Mirshahi

**Affiliations:** ^1^Retina Service, Farabi Eye Hospital, Tehran University of Medical Sciences, Tehran, Iran; ^3^Hamid Riazi-Esfahani: https://orcid.org/0000-0003-2277-398X; ^4^Elias Khalili Pour: https://orcid.org/0000-0001-8123-4375

**Keywords:** Anti-vascular Endothelial Growth Factor, Diabetic Retinopathy, Foveal Avascular Zone, Optical Coherence Tomography Angiography, Panretinal Photocoagulation

## Abstract

**Purpose:**

This study aimed to compare macular vascular changes one and three months after treatment with either panretinal photocoagulation (PRP) or intravitreal bevacizumab (IVB).

**Methods:**

A total of 62 eyes with very severe non-proliferative diabetic retinopathy or early proliferative diabetic retinopathy without center-involved diabetic macular edema, were included in this retrospective study. Thirty-nine eyes were allocated to the PRP group, while 23 eyes were treated with IVB. Optical coherence tomography angiography (OCTA) was performed to measure foveal avascular zone (FAZ) characteristics as well as the densities of superficial and deep capillary plexuses (SCP and DCP).

**Results:**

In the IVB group, the FAZ area and perimeter expanded at month one but returned to baseline level after three months. In the PRP group, however, the FAZ area and perimeter were rather steady. Changes in the FAZ area were significantly different between the treatment groups at month one (*P *= 0.02), but not at month three (*P *= 0.31). There was no significant difference in the change in FAZ circularity index between the two groups at each time point (*P *= 0.55 and *P *= 0.31). Similarly, changes in SCP density were not statistically significant between the two groups at both time points (all *Ps* > 0.05). A comparison of the two treatment arms based on the mean change in DCP density revealed a significant difference at month one, but not at month three (*P *= 0.01 and *P *= 0.49, respectively).

**Conclusion:**

Although bevacizumab and PRP have different short-term macular vascular responses, both therapies have the ability to normalize or stabilize vascular measures over time.

##  INTRODUCTION 

The main established treatments of proliferative diabetic retinopathy (PDR) are panretinal photocoagulation (PRP) and anti-vascular endothelial growth factor (VEGF) injection.^[1]^ For decades, PRP was the mainstay of treatment and was known for preventing severe visual loss in PDR.^[2]^ In the last decade, clinical trials have reported noninferiority of anti-VEGF agents to PRP in treating patients with PDR.^[3,4]^ Less visual field loss and less occurrence/aggravation of diabetic macular edema (DME) have been the benefits of anti-VEGF compared to PRP. Nowadays, clinicians' and patients' preferences determine whether to treat severe non*-*proliferative diabetic retinopathy (NPDR) or PDR with anti-VEGF or PRP.^[1,5]^

Optical coherence tomography angiography (OCTA) is a novel depth-resolved retinal vascular imaging modality, which allows the retinal vasculature to be mapped out at different capillary plexuses. OCTA has the benefit of visualizing the superficial capillary plexus (SCP), deep capillary plexus (DCP), and foveal avascular zone (FAZ) area.^[6]^

The macular vascular density, as well as the FAZ area parameters, are known as effective ways to monitor the progression of diabetic retinopathy (DR).^[7,8]^Click or tap here to enter text. Upon the deterioration of DR, the FAZ may become more irregular and enlarged as a result of capillary occlusion.^[9,10]^Click or tap here to enter text. The published literature reports the possibility of macular vascular alterations after treating PDR patients through PRP or anti-VEGF using OCTA.^[11,12,13,14,15,16,17,18,19]^ A few studies have conducted a direct comparison between these two treatment modalities.^[20,21]^

Of note, these studies have been constrained by the presence of concurrent cystoid macular edema, which could lead to errors in segmentation of the retinal layers.

In this study, we aimed to evaluate and compare very early (one month) and early (three months) course of changes in macular vasculature as well as FAZ area using OCTA in treatment-naïve patients. These individuals had been affected by very severe NPDR or early PDR without center-involving DME and underwent treatment with either PRP or anti-VEGF.

##  METHODS

This retrospective case series was approved by the Institutional Review Board of Farabi Eye Hospital, affiliated with Tehran University of Medical Sciences, Tehran, Iran (IR.TUMS.FARABIH.REC.1400.038). The study protocol adhered to the tenets of the Declaration of Helsinki, and all participants gave written informed consent before entering the study. The recruited individuals consisted of treatment-naïve patients admitted to Farabi Eye Hospital, between September 2021 and February 2022, with very severe NPDR or early PDR without center-involving macular edema. The diagnosis was based on optical coherence tomography (OCT) with a visual acuity of ≥ 0.2 logarithm of the minimum angle of resolution (logMAR) (Snellen: ≤ 20/32). All patients opted to initiate intravitreal bevacizumab (IVB) injections or PRP after receiving information about the advantages and disadvantages of each respective method.

The classification of diabetic retinopathy, ranging from severe NPDR to early PDR, depended on the results of fundus examination, which was performed with the consensus of two retina specialists. Individuals with high-risk PDR were not included in the study due to their potential for vitreous hemorrhage, which would lower the quality of the OCTA images, as well as their higher chance of center-involved DME. Patients with the following criteria were excluded from the study: visual acuity < 20/200 (Snellen), history of treatment with IVB or any kind of photocoagulation, history of ophthalmologic procedures such as cataract surgery or vitrectomy in the past six months, uveitis, uncontrolled glaucoma, presence of exudate or fibrovascular proliferation in the macular area, tractional retinal detachment, vitreous hemorrhage, epiretinal membrane or vitreomacular traction, visible intraretinal cyst in OCT, OCTA images with quality index < 0.4, central macular thickness (CMT) > 320 µm, and refractive error > +3 or < - 3. Eyes with severe media opacity were also omitted due to its potential impact on image quality. Other criteria for exclusion were pregnancy, refusal to sign the consent form, and poor follow-up compliance.

Patients underwent thorough ophthalmic examination including slit-lamp biomicroscopy and dilated indirect ophthalmoscopy. A masked optometrist measured the best-corrected visual acuity (BCVA; Snellen chart), and the results were then converted to logMAR.

### Macular Imaging

For macular vasculature evaluation, the patients underwent OCTA by RTVue XR 100 Avanti instrument (Optovue, Inc, Fremont, CA, USA), which uses a split-spectrum amplitude-decorrelation angiography algorithm to improve the signal-to-noise ratio. After executing the projection artifact removal (PAR) algorithm, the integral module in the Angio Analytics software (version 2017.1.0.151) was utilized to automatically segment the different retinal layers. Manual correction was applied and propagated in case of erroneous determination by the built-in software during follow-ups. The SCP en-face image was segmented with an inner boundary 3 μ m beneath the internal limiting membrane and an outer boundary set at 15 μ m under the inner plexiform layer, whereas the DCP en-face image was segmented with an inner boundary 15 μ m below the inner plexiform layer and an outer boundary 70 μ m beneath the inner plexiform layer.

Recorded parameters were FAZ area, SCP and DCP densities in the 3 × 3 mm image of the center of the macula and central macular thickness (CMT) was measured in the central 1 mm^2^ subfield of the automatic Early Treatment Diabetic Retinopathy Study (ETDRS) grid. Quality score of > 0.4 (according to the built-in RTVue software quality assessment) was accepted for imaging analysis. The built-in software measured and recorded the vascular density in SCP and DCP. The ETDRS grid was used to define the fovea and parafovea while taking into account the 1 mm and 3 mm rings, respectively. The FAZ region of the whole retina slab was automatically estimated in mm^2^ and was double-checked by an experienced investigator.

The circularity index was calculated using the following formula based on the automatically generated perimeter (perimeter of the highlighted FAZ):^[22,23]^

Circularity Index = 4 π × FAZ /Perimeter.

A regular circle has a circularity index of 1. As such, a ratio closer to 0 indicates an irregular shape. All parameters were documented at baseline, month one, and month three after the initial intervention.

It should be added that we excluded eyes with low image quality or various artifacts (including defocus, movement, shadow, and decentration artifacts) from the study preventing precise measurement of vascular density and the FAZ area.

**Table 1:** Best-corrected visual acuity and central macular thickness one month and three months after panretinal photocoagulation or intravitreal bevacizumab injection.


**Parameter**	**Stage**	**Group**		**Diff**	**95% CI**		***P*** **-value † **
		**IVB**	**PRP**	**Lower**	**Upper**	
BCVA (logMAR)	Baseline	Value	0.5 ± 0.22	0.29 ± 0.17	0.20	0.09	0.29	0.01
	Month 1	Value	0.45 ± 0.21	0.31 ± 0.16	0.12	0.02	0.23	0.01
	Change	–0.05 ± 0.2	0 ± 0.09	–0.04	–0.13	0.04	0.31
	*P*-within ‡	0.63	> 0.99		
	Month 3	Value	0.47 ± 0.22	0.34 ± 0.19	0.13	0.16	0.24	0.02
	Change	–0.03 ± 0.19	0.04 ± 0.12	–0.06	–0.15	0.02	0.14
	*P*-within ‡	0.85	0.18		
CMT (µm)	Baseline	Value	255.13 ± 33.16	252.92 ± 24.36	2.02	–14.33	18.37	0.80
	Month 1	Value	258.48 ± 35.41	277.91 ± 43.25	–16.68	–38.99	5.62	0.14
	Change	3.35 ± 17.01	23.57 ± 35.21	–18.53	–33.49	–3.57	0.01
	*P*-within ‡	0.70	0.00		
	Month 3	Value	266.18 ± 29.34	289.57 ± 60.76	–22.00	–54.67	10.66	0.18
	Change	1.55 ± 18.43	34.1 ± 58.37	–30.64	–59.51	–1.77	0.03
	*P*-within ‡	0.99	0.03		
	
	
BCVA, best corrected visual acuity; logMAR, logarithm minimum angle of resolution; CMT, central macular thickness; IVB, intravitreal bevacizumab; PRP, panretinal photocoagulation; CI, confidence interval; Diff, difference *P*-within ‡ : The difference between time points and baseline (change values) in each treatment group; *P* † : The difference between IVB group versus PRP group								

**Table 2:** Foveal avascular zone (FAZ) area, FAZ perimeter, and FAZ circularity index at baseline, one month, and three months after panretinal photocoagulation (PRP) or intravitreal bevacizumab (IVB) injection.


**Parameter**	**Stage**	**Group**		**Diff**	**95% CI**		***P*** ** † **
		**IVB**	**PRP**	**Lower**	**Upper**	
FAZ area (mm ${\;}^{2}$ )	Baseline	Value	0.41 ± 0.14	0.35 ± 0.14	0.05	–0.01	0.13	0.14
	Month 1	Value	0.55 ± 0.34	0.35 ± 0.16	0.20	0.05	0.03	< 0.01
	Change	0.14 ± 0.33	–0.01 ± 0.07	0.15	0.01	0.29	0.02
	*P*-within ‡	0.10	0.99	<@			
	Month 3	Value	0.36 ± 0.14	0.33 ± 0.13	0.03	–0.06	0.13	0.50
	Change	–0.02 ± 0.11	0.02 ± 0.14	–0.04	–0.12	0.03	0.31
	*P*-within ‡	0.79	0.95	<@			
FAZ perimeter (mm)	Baseline	Value	2.7 ± 0.48	2.46 ± 0.47	0.24	–0.06	0.49	0.05
	Month 1	Value	3.19 ± 1.11	2.46 ± 0.58	0.73	0.23	1.23	< 0.01
	Change	0.48 ± 1.12	–0.03 ± 0.29	0.51	0.04	0.98	0.03
	*P*-within ‡	0.10	0.92	<@			
	Month 3	Value	2.48 ± 0.51	2.37 ± 0.59	0.11	–0.27	0.5	0.57
	Change	–0.08 ± 0.46	0.03 ± 0.56	–0.11	–0.45	0.21	0.48
	P-within ‡	0.56	0.95	<@			
FAZ circularity index	Baseline	Value	0.69 ± 0.09	0.71 ± 0.1	–0.01	–0.06	0.03	0.48
	Month 1	Value	0.67 ± 0.11	0.72 ± 0.08	0.04	–0.09	0.01	0.12
	Change	–0.03 ± 0.1	0 ± 0.08	–0.01	–0.06	0.03	0.55
	*P*-within ‡	0.59	0.98	<@			
	Month 3	Value	0.7 ± 0.06	0.72 ± 0.08	–0.01	–0.06	0.03	0.54
	Change	–0.02 ± 0.07	0.02 ± 0.11	–0.03	–0.08	0.02	0.31
	*P*-within ‡	0.98	0.90	<@			
	
	
FAZ, foveal avascular zone; mm, millimeter; IVB, intravitreal bevacizumab; PRP, panretinal photocoagulation; CI, confidence interval; Diff, difference *P*-within ‡ : The difference between time points and baseline (change values) in each treatment group; *P* † : The difference between IVB group versus PRP group								

**Table 3:** Vascular parameters at baseline, one month, and three months after panretinal photocoagulation (PRP) or intravitreal bevacizumab (IVB) injection.


**Parameter**	**Stage**	**Group**		**Diff**	**95% CI**		***P*** ** † **
		**IVB**	**PRP**	**Lower**	**Upper**	
Foveal SCP (%)	Baseline	Value	10.95 ± 4.52	13.52 ± 5.71	–2.67	–5.52	0.18	0.06
	Month 1	Value	10.01 ± 4.52	13.67 ± 5.69	–3.73	–6.69	–0.77	0.01
	Change	–0.94 ± 4.72	–0.33 ± 3.2	–0.56	–2.57	1.44	0.58
	*P*-within ‡	0.69	0.96	<@			
	Month 3	Value	9.76 ± 2.74	14.59 ± 6.6	–4.75	–7.73	–1.77	< 0.01
	Change	–1.4 ± 4.24	1.07 ± 4.77	–1.14	–4.34	1.52	0.34
	*P*-within ‡	0.49	0.59	<@			
Foveal DCP (%)	Baseline	Value	24.17 ± 7.65	26.22 ± 7.09	–2.13	–6.06	1.17	0.28
	Month 1	Value	20.24 ± 7.78	26.28 ± 6.94	–6.53	–10.75	–2.31	< 0.01
	Change	–3.93 ± 7.21	0.15 ± 5.27	–4.13	–7.52	–0.73	0.01
	*P*-within ‡	0.02	0.996	<@			
	Month 3	Value	23.68 ± 5.06	27.74 ± 7.92	–3.98	–8.25	0.28	0.06
	Change	–1.13 ± 6.26	0.44 ± 7.56	–1.57	–6.10	2.94	0.49
	*P*-within ‡	0.99	0.91	<@			
	
	
SCP, superficial capillary plexus; DCP, deep capillary plexus; IVB, intravitreal bevacizumab; PRP, panretinal photocoagulation; CI, confidence interval; Diff, difference *P*-within ‡ : The difference between time points and baseline (change values) in each treatment group; *P* † : The difference between IVB group versus PRP group								

### Interventions

In the PRP group, the guidelines published by the ETDRS research group were followed. Accordingly, laser was administered by an independent ophthalmologist over two consecutive sessions (with an interval of one week); a topical anesthetic was applied. Besides, in each session, 1000 to 1200 gray-white spots (500 µm) were created with an argon laser (532 nm) evenly distributed in all four quadrants.^[24]^ In this study, a surgeon (HRE) conducted all of the PRP laser sessions. The need for additional PRP was investigated at months one and three based on the presence of new neovascularization at the disc or elsewhere or new vitreous hemorrhage with visible fundus. If the CMT increased to more than 310 µm after PRP during follow-ups, IVB was administered.

In the anti-VEGF group, the patients underwent three monthly intravitreal injections of bevacizumab biosimilar (IVB) (StivantⓇ    CinnaGen Co, Iran). This biosimilar drug has been evaluated in previous investigations.^[25,26]^ Intravitreal injections were administered in the operating room under sterile conditions. Topical anesthetic drops were given first and then a lid speculum was inserted. After the application of povidone-iodine 5% into the conjunctival sac for about 1 min, an intravitreal injection of 1.25 mg/0.05 ml (StivantⓇ    ) was performed with a 29-gauge needle (1 ml tuberculin syringes; DispoVan) through the pars plana 4 mm and 3.5 mm posterior to the limbus in phakic and pseudophakic eyes, respectively. All patients received topical chloramphenicol 0.5% four times a day for five days after the injection.

In cases where neovascularization continued to deteriorate following three injections, performing PRP was considered; otherwise, the injections were continued.

### Statistical Analysis

All statistical analyses were performed using the SPSS software (IBM SPSS Statistics for Windows, Version 25.0, released in 2017, IBM Corp, Armonk, NY, USA). The outcomes were reported as mean ± standard deviation (SD). The mean differences between the study groups were analyzed using the generalized estimating equation (GEE) to consider the inter-eye correlation for the enrolled bilateral cases. A *P*-value of < 0.05 was considered statistically significant.

##  RESULTS

A total of 62 eyes of 33 patients were included in the study. Of these, 17 (51.5%) were male, and the mean age of the participants was 59.3 ± 9.1 years. Thirty-nine eyes (21 patients) were allocated to the PRP group, while 23 eyes (12 patients) were treated by IVB. In the PRP group, the neovascularizations were regressed in all eyes with PDR and no eye needed additional laser according to the pre-defined protocol. However, four eyes had CMT greater than 310 µm at the third month (none at one month) and, therefore, underwent IVB injection after final image acquisition. In the IVB group, after three consecutive injections, all neovascularizations were resolved or became barely visible based on fundus examination.

**Figure 1:**
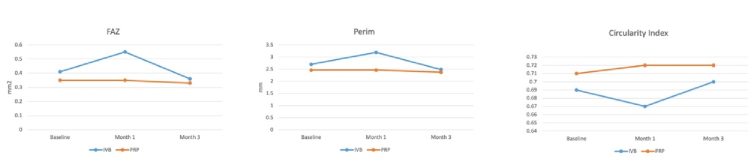
The course of foveal avascular zone (FAZ) area, FAZ perimeter (Perim), and FAZ circularity index during three months of follow-up after intravitreal bevacizumab (IVB) injection and panretinal photocoagulation (PRP).

**Figure 2:**
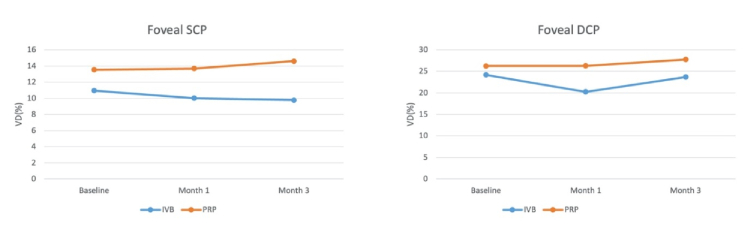
The course of vascular parameters during three months of follow-up after panretinal photocoagulation (PRP) and intravitreal bevacizumab (IVB) injection.

### Best-corrected Visual Acuity (BCVA) and Central Macular Thickness

At baseline, the mean BCVA was significantly different between the IVB and PRP groups (0.5 ± 0.22 vs 0.29 ± 0.17 logMAR, P = 0.01), and this difference remained significant throughout follow-up. Neither group showed significant changes in visual acuity after one and three months [Table 1]. Baseline CMT was similar between groups (IVB: 255.13 ± 33.16 µm, PRP: 252.92 ± 24.36 µm; P = 0.8), however, PRP resulted in a significant CMT increase at months one and three (277.91 ± 43.25 µm, P < 0.01; 289.57 ± 60.76 µm, P = 0.03). IVB did not show significant CMT changes (month 1: 258.48 ± 35.41 µm, P = 0.7; month 3: 266.18 ± 29.34 µm, P = 0.99). Consequently, CMT changes significantly differed between the IVB and PRP groups at both time points (P = 0.01, P = 0.03) [Table 1].

### FAZ Characteristics

At baseline, there was no statistically significant difference in the FAZ area between the two treatment groups (0.41 ± 0.14 mm^2^ in IVB group and 0.35 ± 0.14 mm^2^ in PRP group; *P *= 0.14).

During follow-up, the FAZ area in the PRP group did not change (month one: 0.35 ± 0.16 mm^2^, *P *= 0.99; month three: 0.33 ± 0.13, *P *= 0.95).

The FAZ area increased in the IVB group at month one (0.55 ± 0.34 mm^2^) and then decreased at month three (0.36 ± 0.14 mm^2^), but none of the changes were statistically significant (*P *= 0.10 and *P *= 0.79, respectively). Changes in the FAZ area were significantly different between treatment groups at month one (*P *= 0.02), but not at month three (*P *= 0.31) [Table 2; Figure 1].

The findings on perimeter changes closely resembled those of the FAZ area. While there was no change in the PRP group (2.46 ± 0.47 mm, 2.46 ± 0.58 mm, and 2.37 ± 0.59 mm at baseline, month one, and month three, respectively), the perimeter increased in the IVB group during the first month and then decreased in the third month (2.7 ± 0.48 mm, 3.19 ± 1.11 mm, and 2.48 ± 0.51 mm at baseline, month one, and month three), however, these changes were not significant (*P* > 0.05 for all) [Table 2; Figure 1].

The comparison of the two treatment arms based on the mean change in FAZ perimeter revealed a significant difference at month one, but not at month three (*P *= 0.03 and* P *= 0.48, respectively).

At each time point, neither the PRP group (baseline circularity index: 0.71 ± 0.1, circularity index one month post-PRP: 0.72 ± 0.08, and circularity index three months post-PRP: 0.72 ± 0.08, all *Ps* > 0.05) nor the IVB group (baseline circularity index: 0.69 ± 0.09, circularity index one month post-IVB: 0.67 ± 0.11, and circularity index three months post-IVB: 0.7 ± 0.06, all *P* > 0.05) showed a significant change in the circularity index of the FAZ area. The change in FAZ circularity index did not differ significantly between the two groups at one and three months after treatment initiation (*P *= 0.55 and *P *= 0.31, respectively) [Table 2; Figure 1].

### Vessel Density

At baseline, there was no statistically significant difference in the vessel density (VD) of SCP between the two treatment groups (13.52 ± 5.71 in PRP and 10.95 ± 4.52 in IVB; *P *= 0.06).

The PRP group displayed a significantly higher foveal VD in SCP than did the IVB group at month one (13.67 ± 5.69 vs 10.01 ± 4.52, *P *= 0.013) and month three (14.59 ± 6.6 vs 9.76 ± 2.74, *P* = 0.002). However, none of the changes in foveal VD in SCP at months one and three were statistically significant between the two groups (all *Ps* > 0.05) [Table 3; Figure 2].

Foveal VD in DCP increased marginally following PRP, but this increase was not statistically significant (baseline: 26.22 ± 7.09, month one: 26.28 ± 6.94, and month three: 27.74 ± 7.09, all *Ps* > 0.05). In contrast, the foveal VD in DCP decreased significantly in the IVB group after one month (baseline: 24.17 ± 7.65, month one: 20.78 ± 7.78, *P* = 0.023).

Based on the between-group analysis, the IVB group exhibited a significantly lower foveal VD in DCP after one month (20.24 ± 7.78 vs 26.28 ± 6.90, *P* = 0.001). However, the foveal DCP increased and returned to its initial value at month three (month three: 23.68 ± 5.06, *P *= 0.99).

Comparing the two treatment arms in terms of the mean change in DCP density revealed a significant difference at month one, but not at month three (*P *= 0.01 and *P *= 0.49, respectively) [Table 3; Figure 2].

##  DISCUSSION

An increasing number of studies have assessed the anti-VEGF injection as a potential alternative to the conventional PRP in PDR.^[1,4]^ In the current study, we used OCTA to compare the very early (one month) and early (three months) changes in macular vascular parameters following PRP and IVB injections in patients with very severe NPDR and early PDR.

In the very early follow-up (one month), the initiated treatments (IVB vs PRP) entailed statistically significant differences in several vascular parameters on OCTA (i.e., FAZ area, FAZ perimeter, and foveal DCP density). Interestingly, all of these differences became non-significant in the third month following therapy.

Different treatment modalities (IVB vs PRP) may have different effects on the FAZ area. In the current study, at one month, the FAZ area and perimeter changes were substantially different across the two treatment groups (both Ps = < 0.01). After the initiation of IVB injections, the FAZ area and perimeter expanded modestly at month one but returned to baseline level after three months. In the PRP arm, however, FAZ area and perimeter were rather steady.

Two previous studies have observed that the FAZ area remained stable for just one month after a single intravitreal anti-VEGF injection in patients with center-involved DME.^[15,19]^ In another study on 40 eyes, three monthly IVB injections for DME resulted in a significant increase in the FAZ area.^[12]^ However, all of these studies have been limited by concurrent macular cystic edema, which may cause segmentation mistakes and, in particular, inconsistent segmentation selection due to the variety in size and location of cysts with anti-VEGF therapy. One of the strengths of the current study is that individuals with cystoid macular edema were eliminated from the study.

Abdelhalim et al^[27]^ showed that PRP dramatically improved the FAZ area; it decreased from 0.56 ± 0.27 mm ${\;}^{2}$ at baseline to 0.50 ± 0.21 mm^2^ after one month and 0.46 ± 0.21 mm ${\;}^{2}$ after six months. On the other hand, Kim et al^[28]^ demonstrated that there was no substantial change in the FAZ area after PRP, which is consistent with the results of the current study.

In the current study, the foveal SCP and DCP dropped one month after IVB injection, although only the decline in DCP density was significant (P = 0.02). The SCP and DCP values at month one (both P = 0.01) and month three (P = 0.01 and P = 0.06, respectively) were lower in the IVB group compared with the PRP group. Indeed, in the first month, DCP density showed a significantly higher reduction in the IVB group than the PRP group (P = 0.01), which was compatible with FAZ enlargement in the IVB group. However, both changes (FAZ enlargement and DCP density reduction) were compensated three months following the first IVB injection. Using OCTA, Zhao et al compared the effect of intravitreal conbercept with PRP on macular microvasculature in PDR eyes. They noticed no significant changes in macular VD between the treatment modalities 12 months after intervention, although they did not evaluate the early changes.^[20]^ On the contrary, Li et al found that over an average follow-up period of two years, higher foveal capillary densities in both SCP and DCP were observed in the eyes treated with PRP compared with those eyes that received conbercept.^[21]^ In the post hoc analysis of a recovery study on patients with PDR, the macular VD of SCP and DCP did not show a significant change after monthly or quarterly intravitreal injections of aflibercept for 12 months.^[29]^ The researchers, accordingly, suggested that even a monthly treatment of anti-VEGF drugs does not affect macular VD in the long term. Some studies have reported the vasoconstriction of retinal arterioles due to a transient reduction of nitric oxide (NO) in the early stages following anti-VEGF therapy.^[30,31]^ Theoretically, this transient vasoconstriction should result in a decrease in VD and FAZ enlargement, especially in DCP which is more vulnerable to ischemia.^[30,32]^ Nevertheless, no evidence exists to indicate the endurance of this vasoconstriction, as demonstrated by the aforementioned studies. The authors of the present study assume that retinal microvascular intrinsic autoregulation could potentially counterbalance this temporary decrease in vascular density. Therefore, it may not affect the vascular densities and FAZ area at month three as we observed in the current study. Nevertheless, it is unlikely that anti-VEGF therapy would exacerbate macular ischemia in DR eyes. The stability of VD throughout anti-VEGF therapy suggests that this treatment approach may be helpful for patients with macular nonperfusion, which is worthy of mention because nonperfusion is presumed to worsen in individuals with diabetic retinopathy.^[20,29]^

We noted a reducing trend in SCP and DCP densities after IVB injection (particularly at one month), but an increasing trend following PRP. Similarly, Fawzi et al^[14]^ suggested a general shift of blood flow to the posterior pole after PRP using an adjusted flow index (a self-created surrogate metric of blood flow) for six months, however, they did not detect a significant change in vascular density measures. Using the FAZ circularity index, we previously emphasized this redistribution tendency six months after PRP.^[13]^ As the patients were just followed up for three months, we did not detect any alteration in the circularity index of the FAZ. We assume the etiology of this foveal and parafoveal limited flow redistribution might be explained by the transient reperfusion of occluded vessels after PRP due to inflammatory mediators and NO overproduction.^[14,33]^ Similar to our study, the increase in vascular densities after PRP has not been significant over time in previous investigations. In other words, the short-term (one month) PRP-induced VEGF overexpression is compensated by improved oxygenation of the retina and the resulting reduction in VEGF production in the long term.^[34]^ Moreover, this stability or increasing trend after PRP is counterbalanced by an overall trend for decreased capillary density along with a significant increase in capillary nonperfusion in treatment-naïve patients with DR.^[14]^

There are several limitations to our research results. The main shortcomings are the small sample size and the relatively brief follow-up period, making it difficult to draw definitive conclusions about the relationship between changes in vascular parameters and the administered therapeutic methods. Also, shortly after the injections, the retinal microvascular structure in the IVB group may have experienced more significant impacts that could not be identified when assessing changes after one month. Investigations on pharmacokinetics of bevacizumab have shown that although vitreous concentrations of bevacizumab decline in a monoexponential fashion with a half-life of 4.32 days, concentrations of > 10 microg/ml bevacizumab are maintained in the vitreous cavity for 30 days.^[35]^ Another study has shown that vitreous VEGF concentrations decrease to < 31.2 pg/mL, the lower limit of detection, between 1 and 28 days after injection; but they return to the pre-injection level at 42 days.^[36]^ This study has focused on eyes devoid of significant macular edema and having very severe NPDR or PDR without high-risk characteristics. As a result, the findings cannot be applied to eyes with high-risk PDR or macular edema. Another limitation of the present study is the lack of data on metabolic management, including hemoglobin A1c (HbA1c), lipid profile, and blood pressure, which are known to play a major role in the course of DR. It has been shown that high glucose, as well as insulin administration, can alter vessel diameter and flow speeds in the retinal vasculature.^[20]^ Additionally, we used a biosimilar bevacizumab for intravitreal injections. If the original medicine or another anti-VEGF could be injected, the outcomes might differ. Finally, we only performed a macular region scan (3 × 3 mm scans).

Recently, widefield swept-source OCTA has become available to evaluate capillary perfusion of the midperiphery in addition to the posterior pole. The distinction in retinal microvasculature influenced by PRP and anti-VEGF treatments is likely to become more pronounced in upcoming years, as researchers investigate significantly larger scanned retinal areas and sample sizes in randomized longitudinal prospective studies with extended follow-up periods.

In summary, the OCTA results indicated statistically significant differences in several vascular parameters (i.e., FAZ area, FAZ perimeter, foveal DCP vascular density) between the two types of therapies (IVB vs PRP) one month after starting treatment. All of these effects, however, faded to insignificance in the third month after treatment. Larger-scale randomized controlled trials are needed to validate and justify the findings of this study.

### Financial Support and Sponsorship

None.

### Conflicts of Interest

None.
